# Location In Vivo of the Innervation Zone in the Human Medial Gastrocnemius Using Imposed Contractions: A Comparison of the Usefulness of the M-Wave and H-Reflex

**DOI:** 10.3390/jfmk7040107

**Published:** 2022-11-28

**Authors:** Rodrigo A. Guzmán-Venegas, Felipe H. Palma-Traro, Oscar D. Valencia, María José Hudson, Patricio A. Pincheira

**Affiliations:** 1 LIBFE Laboratory, School of Kinesiology, Faculty of Medicine, Universidad de los Andes Chile, Santiago 7620086, Chile; 2 School of Human Movement and Nutrition Sciences, Faculty of Health and Behavioural Sciences, The University of Queensland, Brisbane 4072, Australia; 3 School of Health and Rehabilitation Sciences, Faculty of Health and Behavioural Sciences, The University of Queensland, Brisbane 4072, Australia

**Keywords:** innervation zones, neuromuscular junction, M-wave, H-reflex, imposed contractions, electrical stimulation, gastrocnemius muscle

## Abstract

The anatomical territory where the neuromuscular junctions are grouped corresponds to the innervation zone (IZ). This can be located in-vivo using high-density electromyography and voluntary muscle contractions. However, in patients with motor impairment, the use of contractions imposed by electrical stimulation (ES) could be an alternative. The present study has two aims: Firstly, to describe the location of the IZ in-vivo of the medial gastrocnemius (MG) using imposed contractions by ES. Secondly, to compare the usefulness of M-waves and H-reflexes to localize the IZs. Twenty-four volunteers participated (age: 21.2 ± 1.5 years). ES was elicited in the tibial nerve to obtain M-waves and H-reflexes in the MG. The evaluators used these responses to localize the IZs relative to anatomical landmarks. M-wave and H-reflex IZ frequency identification were compared. The IZs of the MG were mostly located in the cephalocaudal direction, at 39.7% of the leg length and 34% of the knee’s condylar width. The IZs were most frequently identified in the M-wave (83.33%, 22/24) compared to the H-reflex (8.33%, 2/24) (*p* > 0.001). Imposed contractions revealed that the IZ of the MG is located at 39.7% of the leg length. To locate the IZs of the MG muscle, the M-wave is more useful than the H-reflex.

## 1. Introduction

Muscle fibers are innervated by alpha motor neurons (αMN) through neuromuscular junctions. Topographically, neuromuscular junctions are grouped within the muscular architecture, specifically in the so-called innervation zone (IZ) [1,2,3,4,5]. Locating the IZ is useful, for instance, to define the best place to position electrodes for the electrophysiological study of the muscle [6,7]. Also, it could help to identify the site for a botulinum toxin injection in order to improve its efficacy in the treatment of spasticity or dystonia [8].

A non-invasive technique that facilitates the localization of the IZ in vivo of different muscles is the electromyogram obtained with high-density surface electromyography (HD-sEMG). This procedure requires voluntary muscle contractions (VC), usually in levels varying between 10 and 75% of the maximum force [9,10]. However, this may not be applicable in those patients who may have low voluntary muscle activation, which is also usually associated with a lower amplitude of the electromyographic recordings of the affected muscles [11]. An alternative for the location of the IZ in vivo without VCs might be the use of involuntary contractions elicited by electrical stimulation (ES). After ES of a mixed nerve, two evident muscle activations are observed on the electromyogram: the Hoffman reflex (H-reflex), which is a consequence of the depolarization of muscle afferents, and the M-wave, which is produced as a result of direct stimulation of motor fibers [12,13]. Both waves are the consequence of motor unit (MU) activation and are potentially useful for the localization of IZs. However, the degree of usefulness that both waves provide to identify the IZ is unknown.

In the lower extremities, a large number of studies have focused on the H-reflex of the gastrocnemius muscle [13,14] due to the easy access to the tibial nerve for ES and the importance of this muscle group in both daily activities (e.g., walking, standing up from a seated position, etc.) and sports (e.g., running, jumping, etc.). It is also, for example, a muscle that is frequently affected by neurological conditions such as post-stroke spasticity [15] and commonly treated with botulinum toxin infiltration [8]. Indeed, IZs have previously been located in the MG using voluntary contractions [5,16,17]. Despite this, there are no studies that have described the location of the IZs of the MG in vivo using contractions imposed by ES. In this way, the present study intended to provide new information about the location of the IZs of the MG, using a method that is independent of the ability that a person may have to generate VCs. The present study has two aims. First, describe the location of the IZ in vivo of the medial gastrocnemius (MG) muscle using imposed contraction by ES. Second, compare the usefulness of M-waves and H-reflexes to localize the IZs of the MG muscle.

## 2. Materials and Methods

### 2.1. Volunteers

Twenty-four healthy volunteers participated in the study (men: 14; women: 10; age: 21.2 ± 1.5 years, weight: 67.4 ± 13.2 kg, height: 1.68 ± 0.80 m). This sample was recruited according to the following exclusion criteria: obesity (body max index > 30), history of lower extremity injuries in the last six months, central or peripheral neurological diseases, injury or infection of the skin in the area of the MG and having any contraindication for the use of ES [18]. All volunteers gave their consent in writing. The procedures used in this study were in accordance with the Declaration of Helsinki of 1975 and were approved by the local Bioethical Committee (Ethical Application: CEC202023).

### 2.2. Instrumentation

**Figure 1:**
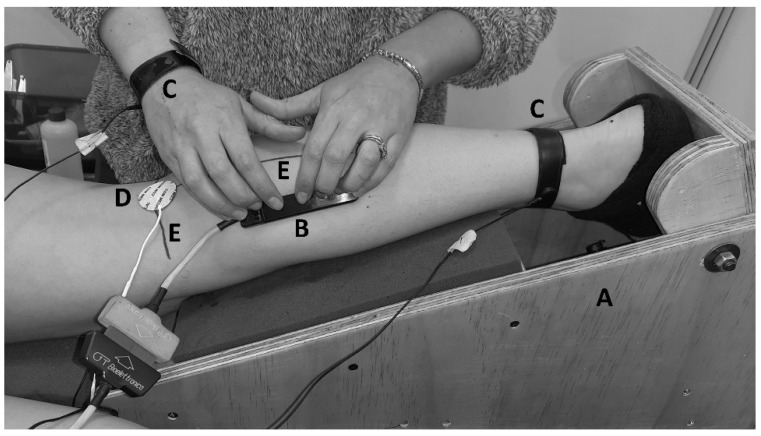
Setup. (**A**) The ankle was fixed with a custom-made modular wood brace so that the ankle joint was kept at 15° of plantarflexion. (**B**) Semi-rigid linear array of 16 electrodes with a silver 5 mm inter-electrode distance. (**C**) Reference for electromyography. (**D**) Cathode for electrostimulation. (**E**) Landmark anatomical frame.

**Figure 2:**
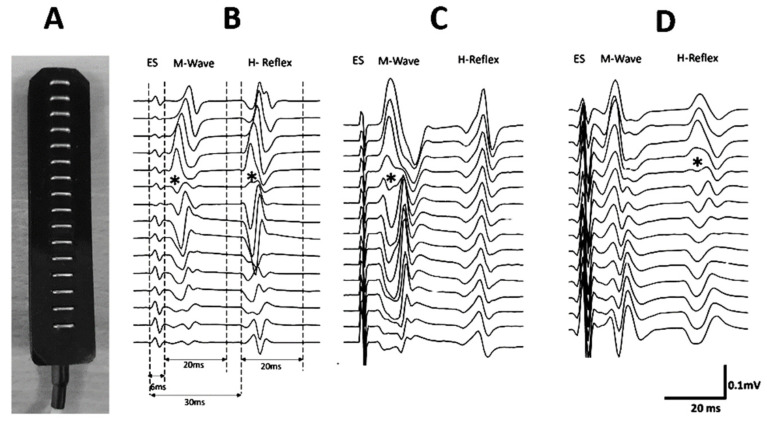
A linear array of 16 electrodes was used (**A**). Examples of locations of innervation zones (IZ) of the medial gastrocnemius muscle for one volunteer. The innervation zones are shown with (*) (**B**). The windows in which the EMG amplitudes of the M-wave and H-reflex were calculated are shown. (**C**) An IZ is observed in the M-wave but not in the H-reflex. (**D**) An IZ is seen in the H-reflex but not in the M-wave.

For the HD-sEMG acquisition, a linear array of 16 electrodes (silver rods of 1 × 5 mm, SA 16/5, OTbiolettronica, Torino, Italy, Figure 1 and Figure 2A) with an inter-electrode distance of 5 mm was used. The EMG signals were amplified in a single differential mode (EMG-USB2, OT Biolettronica, with a sampling frequency of 2048 Hz, 3 dB bandwidth 10–500 Hz, gains of 1000–2000). Electrical stimuli (Single pulses, 1ms duration) were applied with an electrical stimulator (FES-4 OTP, TrainFes, Biomedical Devices Spa., Santiago, Chile). All signals were recorded and stored using data acquisition software (OT Biolab+.V1.5.5.0 OT Biolettronica, Torino, Italy).

### 2.3. Procedure

In a controlled and quiet environment [13], each volunteer was positioned on a bench in the prone position with their arms next to their trunk. With the right knee at 20° flexion (0° full extension), the right leg and foot were placed in a custom-made modular wood brace used to fix the ankle joint at 15° of plantar flexion. For EMG recordings, the skin over the area above the MG muscle was prepared by shaving, cleansing with an abrasive paste (Everi, SpesMedica, Genoa, Italy), and washing with water. An evaluator then drew two reference lines, the first on the popliteal fold (*X*-axis: knee width), and the second between the midpoint of the popliteal fold and the distal insertion of the calcaneal tendon (*Y*-axis: leg length) (Figure 1). These lines were considered as the anatomical landmark frame (ALF) [19]. A second evaluator placed two adhesive electrodes (CDM Medical Spa, Las Condes, Chile) for the ES: The anode (5 × 5 cm) was positioned superior to the patella, and the cathode (radius 15 mm) was fixed on the popliteal fossa. For the M-wave and H-reflex recording, an evaluator placed the linear electrode array parallel to the *Y*-axis of ALF (Figure 1). The evaluator repositioned the electrode array until an adequate propagation of compound muscle action potential (CMAP) was visually recognized. When adequate CMAP propagation was achieved (Figure 2B–D), single pulses of progressively increasing intensity (from 2 mA to the maximal M-wave in 1 mA increments) were delivered to the tibial nerve. Ten-second rests were given between stimulation at each intensity to avoid potential confounding post-activation depression [20].

The location of the IZ was defined in vivo based on the following criteria (Figure 2B–D): location where an inversion of CMAP was observed or where a minimum amplitude signal was registered between two inverted CMAP [3,4,5,17,21,22,23]. Two evaluators participated in the identification of the IZs. Once the IZs were located, they were marked on the skin, and XY coordinates based on the ALF were assigned. IZ coordinates were expressed in millimeters (absolute positions) and relative to the length of the respective axes of the ALF as percentages (adjusted positions). During electrostimulation, the electromyographic activity of the MG was recorded and stored for the comparison of the usefulness of M-wave and H-reflex IZ location.

### 2.4. Signal Analysis

All stored EMG signals were filtered offline with a second-order Butterworth digital filter with a bandwidth of 20–400 Hz (OT Biolab+.V1.5.5.0 OT Biolettronica, Torino, Italy). The visualization of the electromyogram and M-wave and H-reflex processing for IZ identification was done using custom-made scripts written in a signal processing software (IgroPro 9.0, WaveMetrics Inc., Portland, OR, USA). The amplitude of the M-wave and H-reflex was calculated using peak-to-peak estimations. For the M-wave detection, a window of 20 ms duration was defined as 6 ms after the onset of the artifact produced by the electrostimulation. For the H-reflex detection, a window of 20 ms was defined as 30 ms after the onset of the artifact produced by the electrostimulation [13,24] (Figure 2B). The IZs in the M-wave and H-reflex of each volunteer were identified within a time window of 50 ms after the electrical stimulus (Figure 2B). This window corresponds to the electrical stimulus with which amplitudes were maximal for M-wave and H-reflex (M-wave Max and H-reflex Max).

Three evaluators participated in the comparison of the usefulness of M-waves and H-reflexes to localize the IZs in the MG muscle. Usefulness was defined as the frequency with which each wave (M-wave or H-reflex) allowed the identification of the IZs. This was done by each evaluator in a blind manner with respect to their peers. Each evaluator wrote down in his personal 24 × 2 (24 volunteers x M-wave, H-reflex) form a “1” when the IZ was identified and a “0” when it was not identified. The order in which the windows of each volunteer were examined was the same for each evaluator. Consecutively, the data from the first and second evaluators were compared. When there was a discrepancy between them, the record of the third evaluator was used to resolve the differences. In this way, from the three evaluators, a single form was obtained, containing the distribution of 1 and 0, for the M-wave and H-reflex wave of the 24 volunteers. In order to consider the identification of the IZs valid, it was defined a priori that the level of agreement between the evaluators should be at least close to perfect [25] (kappa index > 0.80).

### 2.5. Statistical Analysis

In order to describe the location of the IZ of the MG, the absolute and adjusted X and Y coordinates were first analyzed using a D’Agostino & Pearson test in order to determine if their distribution complied with the assumption of normality. From this, the ad hoc measures of centralization and dispersion were used. In order to determine which of the waves proved to be more useful for locating IZs, the IZ identification frequencies from M-wave and H-reflex were compared using a Chi-square test. The sample size was determined considering an inter-rater agreement analysis based on the recommendations of Tractenberg et al. [26]. Considering a statistical power of 80%, an alpha of 5%, and assuming a kappa coefficient > 0.8 (k1 = 0.3, k2 = 0.8) for an analysis of two categories (presence/absence of IZ), the minimum sample size to detect concordance between the evaluators was 23 participants. The concordance between the evaluators was determined using Cohen’s kappa coefficient. This statistical test was used since it allows the inter-rater reliability for categorical variables to be measured in a more robust way than the percentage of agreement [27]. The concordance between evaluators was calculated for IZ localized using M-waves and H-reflexes. All statistical analyses were done with a confidence level of 95%, and a *p*-value lower than 0.05 was considered to determine statistical significance. Statistical analyses were performed using STATA software (STATA/SE 12.1, StataCorp LP, College Station, TX, USA).

## 3. Results

The absolute and adjusted positions of the IZs of the MG obtained through imposed contractions are shown in Table 1. Examples of the IZs located by imposed contractions are shown in Figure 2. Both positions, adjusted and absolute, did not show normal distributions (*p* < 0.05). Therefore, the positions of the IZs were represented by medians and their interquartile ranges (IR) (Table 1). Regarding the usefulness of M-waves and H-reflexes for detecting the IZs, it was found that in 83.33% (20/24) of the cases, the IZ was identified from the M-wave, while in only 8.33% (2/24) of the cases, the IZ was observed in the H-reflex. The Chi-square analysis indicated a significant difference (*p* < 0.001) when detecting the IZs based on the M-wave vs. H-reflex. The global level of agreement (using both M-wave and H-reflex) between the evaluators for the detection of the IZ was 97.92%, with a kappa index of 0.96 [0.88–1.00]. The level of agreement between the evaluators for the detection of the IZ was 95.83%; kappa = 0.86 [0.60–1.00] and 100.00%; kappa = 1.00 [1.00–1.00], for M-wave and H-reflex, respectively.

## 4. Discussion

In the present study, the IZs of MG could be located by contractions imposed by ES. The IZs of the MG were located in the cephalocaudal direction at 39.7% (IR: 36.5–42.8) of the ALF. Our results are similar to those reported previously by Beretta Piccoli et al. [16] using voluntary contractions. These authors reported the location of IZs at 41% of the ALF (no IR reported). However, our results are somewhat different from Barbero et al. [17], who used voluntary contractions to report the location of the IZ of MG at 34% (IR: 29–40) of the ALF. Apart from the type of contraction used, some methodological differences could explain the discrepancy between our results and those previously reported. The joint position is a determining factor in the location of the IZs relative to the ALF [28]. Displacements of 5–30 mm of IZ as a result of the joint position were found [28]. In previous studies, the joint position variable was not controlled instrumentally [16,17], while in the present study, this variable was controlled. Thus, the ankle was fixed by a modular wood brace (Figure 1), which possibly involved a minimal shortening of the muscle fiber. The relative similarity of the results obtained using imposed contractions and the available data obtained with VCs suggest that the use of the contractions imposed by ES could be an alternative procedure for locating the IZs of MG, as has been demonstrated in the anterior tibial muscle [19].

Few studies have used ES in conjunction with HD-sEMG for the localization of the IZs. Zhang et al. used the M-wave to localize the IZs in the biceps brachii by stimulation of the musculocutaneous nerve [29,30]. Guzmán-Venegas et al. located the IZs of the anterior tibialis muscle using M-wave evoked by ES. However, in these studies, the H-reflex was not considered. The results of the present research show that M-waves and H-reflexes, recorded in the same muscle location, do not have the same capacity to identify the IZs of the MG. Our results indicate that the IZs are observed with a higher frequency in the M-wave compared to the H-reflex (*p* < 0.001). The agreement registered between evaluators was almost perfect [25]. This agreement was assessed for both detection and non-detection of IZs. The inter-rater agreement was higher for the H-reflex (kappa = 1.00) compared to the M-wave (kappa = 0.86). However, this concordance is mainly associated with the cases where no IZs were detected because, with the H-reflex, it was only possible to identify the IZs in 8.3% of the cases (2/24).

The different capacities to show the IZs through the M-wave and H-reflex could be attributed to the neurophysiological mechanisms involved in the production of these waves. On the one hand, the M-wave is a product of electrical stimulation of the motor fibers of the peripheral nerve, causing a direct activation of the efferent fibers, sending an action potential directly from the application point to the neuromuscular junction, producing the activation of the MU. As this is a direct activation, few variables modulate this response, including the intensity of the electrical stimulus and the activation threshold of efferent fibers [13,31]. On the other hand, the H-reflex is produced by the same electrical stimulation of the peripheral nerve but, as a result, obtains an action potential that travels along the afferent fibers reaching the synapse with the αMN. This produces an action potential of the efferent pathways generated by the αMN, which is transmitted until it reaches the neuromuscular conjunction producing a twitch response on the electromyogram. Since spinal neural circuits are involved in the generation of the H-reflex, there are numerous variables that could influence it, for example, pre-synaptic inhibition, post-activation depression, and motoneuron excitability [32]. It has also been identified that joint position [33], the length of the studied muscle [34]; the presence or absence of movement [35]; the position of the head [36]; the contraction of other muscle groups [37], the position of the extremities [37,38,39] and the exposure to a noisy environment [13], affect the characteristics of H-reflex. Therefore, the H-reflex may be less useful for identifying and locating IZ. Another reason why the H-reflex is less useful for detecting IZ compared to the M-wave could be the way MU recruitment occurs in each wave type. Since the H-reflex originates from central circuits at the spinal level, it could recruit the MUs in a more synchronous way, thereby increasing the interference between MUAPs of the activated MUs [40], masking the location of IZs, as seen in Figure 2C.

A limitation of the present study is the consideration of a single EMG recording site in the MG; future studies could consider whether the frequency of IZ recognition is based on M-wave or H-reflex changes depending on the muscle region evaluated. Based on the results obtained in this study, it could be hypothesized that there should be no differences between the locations of the IZs, obtained by VCs and imposed by ES. Future studies should compare the concordance obtained from the localization of the IZs of MG in VCs versus those imposed by ES.

## 5. Conclusions

From the results obtained in this research, in the evaluated sample, it can be concluded that imposed contractions revealed that the IZ of the MG is located at 39.7% of the leg length. To locate the IZs of the MG muscle, the M-wave is more useful than the H-reflex.

## Figures and Tables

**Table 1:** Absolute and Adjusted position of innervation zones of the medial gastrocnemius muscle (n = 24). Data are presented as medians and interquartile ranges.

	Absolute Position		Adjusted Position		Anthropometry	
	X (mm)	Y (mm)	X (%)	Y (%)	Knee Width (mm)	Leg Length (mm)
Median	33.0	165.0	34.0	39.7	94.5	406.5
25% Percentile	20.3	144.5	23.0	36.5	91.3	395.3
75% Percentile	45.0	177.0	48.5	42.8	98.5	422.3

X (%): X-coordinate of the innervation zone adjusted to the condylar width of the knee. Y (%): Y-coordinated of the innervation zone adjusted to the length of the leg.

## Data Availability

Data are available upon appropriate request.
